# Heart rate variability among women undergoing *in vitro* fertilization treatment: Its predictive ability for pregnancy

**DOI:** 10.1371/journal.pone.0193899

**Published:** 2018-03-12

**Authors:** Meng-Hsing Wu, Pei-Fang Su, Kuan-Ya Chen, Tung-Hee Tie, Hsu-Cheng Ke, Hau Chen, Yu-Chi Su, Yu-Chen Su, Huang-Tz Ou

**Affiliations:** 1 Division of Obstetrics and Gynecology, Department of Internal Medicine, National Cheng Kung University Hospital, Tainan, Taiwan; 2 School of Medicine, College of Medicine, National Cheng Kung University, Tainan, Taiwan; 3 Department of Statistics, National Cheng Kung University, Tainan, Taiwan; 4 Institute of Clinical Pharmacy and Pharmaceutical Sciences, College of Medicine, National Cheng Kung University, Tainan, Taiwan; 5 Department of Pharmacy, College of Medicine, National Cheng Kung University, Tainan, Taiwan; 6 Department of Pharmacy, National Cheng Kung University Hospital, Tainan, Taiwan; Pondicherry Institute of Medical Sciences, INDIA

## Abstract

**Objective:**

This study aimed to assess predictive ability of heart rate variability (HRV) for pregnancy outcomes with in vitro fertilization (IVF) treatment.

**Research design and method:**

A total of 180 women with 261 cycles of IVF and 211 embryo transfers (ETs) were analyzed. HRV was measured at four times during IVF treatment: the first date of menstruation, r-HCG (Ovidrel) administration, and before and after ET. Pregnancy indicators included chemical pregnancy, ongoing pregnancy (> 10 weeks), and live birth (pregnancy > 24 weeks). Mixed effect models were applied to identify predictors for IVF pregnancy. The area under the receiver operating characteristic curve (AUC) was used to assess prediction models for pregnancy.

**Results:**

The HRV values increased during IVF treatment and then decreased after ET. The trend of changes in HRV values during IVF treatment was significant among patients with chemical pregnancy (*p* < 0.01) and those with live birth (*p* = 0.02). Women without pregnancy had lower HRV compared to those with IVF pregnancy (*p* < 0.05). With a one unit increase in HRV difference before and after ET, the odds of chemical pregnancy decreased by 18% (odds ratio; OR: 0.82, 95% CI: 0.70–0.97, *p* < 0.02). With a one year increase in maternal age, the odds decreased by 16% (OR: 0.84, 95% CI: 0.76–0.93, *p* < 0.01), 25% (OR: 0.75, 95% CI: 0.58–0.93, *p* = 0.02), and 28% (OR: 0.72, 95% CI: 0.54–0.91, *p* = 0.01) for chemical pregnancy, ongoing pregnancy, and live birth, respectively. The AUCs were 0.77 (95% CI: 0.70, 0.84), 0.89 (0.79, 0.98), and 0.91(0.83, 0.99) for the prediction models for chemical pregnancy, ongoing pregnancy, and live birth, respectively.

**Conclusions:**

Reduced HRV may be an indicator for low chance of IVF pregnancy. The changes in HRV before and after ET and maternal age might be prognostic predictors of IVF pregnancy.

## Introduction

Undergoing in vitro fertilization (IVF) treatment is psychologically and emotionally stressful for most patients, with perceived distress, depression, or anxiety possibly present before, during, and/or after IVF treatment [1–3]. The fear of not getting pregnant, cost of IVF treatment, daily injections, required procedures, and possibility of failure at any stage of the treatment are the sources of stress. These mental symptoms are believed to negatively impact fertility [3]. The psychological stressors present may impact the hypothalamic pituitary adrenal axis, sympathetic nervous system, and major “fight or flight” stress hormones, which affect women’s heart rate variability (HRV).

Heart rate and rhythm are largely controlled by the autonomic nervous system [4]. HRV is a measure of beat-to-beat alterations in heart rate and serves as an indicator of autonomic nervous system tone. HRV has been shown to be a reliable and non-invasive measure for quantifying cardiovascular autonomic regulator responses to autonomic regulatory mechanisms [5–11]. A spectral analysis of short-term recordings under controlled conditions allows the identification of those rhythmic components at low (LF) and high (HF) frequency known to reflect autonomic modulation of sinus node. Although HF can be a noninvasive index of parasympathetic modulation, and LF mainly reflects sympathetic modulation, a vagal contribution cannot be excluded [5–11]. As a result, the ratio of LF to HF has been proposed and used as a noninvasive index of ‘‘sympathovagal balance.”[7, 8, 11]

HRV has been utilized for diagnosing cardiovascular diseases [12, 13], with reduced HRV associated with worse cardiovascular outcomes. Also, reduced HRV is related to mental symptoms such as generalized anxiety disorders and major depression [14]. Thoughts, emotions, and external experiences are intertwined with the rhythm of the heart and breathing. Constant acute stress, aging, and physical inactivity can lower HRV. Hence, HRV is considered to be a psychophysiological marker of physical and mental well-being.

Only two studies have applied HRV to female fertility. One compared women with a history of unexplained pregnancy loss to healthy females [15] and the other compared women with IVF pregnancies to those with normal pregnancies [16]. These studies only provided a descriptive pattern of HRV among these high-risk women. There is a lack of analytic studies on whether HRV as a psychophysiological marker of physical and mental well-being can be used as a prognostic indicator for pregnancy outcomes for women receiving IVF treatment. The present study assesses the association between HRV and the pregnancy outcomes of women on IVF treatment based on progressive phases (i.e., chemical pregnancy, ongoing pregnancy, live birth). We were particularly interested in the predictive ability of HRV and other influential factors (e.g., maternal age) for pregnancy outcomes with IVF treatment.

## Material and methods

This was a prospective observational study. Before commencement of the study, permission was obtained from the Institutional Review Board (IRB) of National Cheng Kung University Hospital, Tainan, Taiwan (B-ER-105-114).

### Participants

All participants with infertility problems and undergoing embryo transfers (ETs) were recruited from the assisted reproductive technology center at National Cheng Kung University Hospital from January, 2014, to December, 2015, and those with poor quality of embryo were excluded from analysis. A total of 180 women with 254 cycles of IVF and 211 ETs were included in this study. For those with lost follow-up after enrollment, the observation on them was censored (stopped). Data were collected based on the examinations and medical history of the participants. The clinical data at the time of HRV measurements included age, history of gravida and parity, infertility duration, infertility factor (i.e., male, female, both, unknown), and history of pregnancy loss. Since all procedures and treatments were routine care and the patients’ data were analyzed anonymously, the form of consent to participants was waived by the IRB committee.

### HRV measurement

During IVF treatment, study participants received an HRV examination at the following four times: at first date of menstruation (annotated as HRV_D), r-HCG (Ovidrel) administration (annotated as HRV_BO), within one hour before ET (annotated as HRV_BET), and within 2~3 hours after ET (annotated as HRV_PET). All participants were required to rest for 10 min before an examination was performed. During examination, the participants rested in the supine position at room temperature (25±1°C) in a comfortable environment. The WSB Treatment System (WSB-100, Shine Alpha Electronic Co. LTD, Taiwan, R.O.C.), a machine using the time-domain method, was used to record surface electrocardiography (ECG) for 5 minutes. Since a 5-minute ECG recording was taken, the HRV data in the present study were considered as short-term measures. On continuous ECG, each QRS complex from lead V1 and V5 was detected, and the normal to normal (N-N) intervals were recorded. Standard deviations of N-N intervals (SDNN), which is the main variable in the time-domain analysis of HRV, were calculated automatically by the WSB treatment system. Respiratory rhythm is not further measured in our study.

The results of the time-domain analysis were then automatically converted to frequency-domain data using Fourier transformation to obtain the low-frequency/high-frequency (LF/HF) ratio [17], which was used as the HRV value in this study. The LF (0.04–0.15 Hz) and HF (0.15–0.4 Hz) components in the frequency-domain data were defined and obtained from the power spectra obtained from spectral analysis. The HF value represents the activity of the parasympathetic nervous system, as it is almost entirely regulated by it [11, 17], and the LF value represents the sympathetic nervous system activity, as it reflects the modulation of both sympathetic and parasympathetic nervous systems [11, 17]. The LF/HF ratio thus indicates the autonomic nervous system activity of a patient. Normal LF/HF ratios are in the range of 0.5–2.5 [18] that is generally considered optimal for autonomic nervous system balance.

### Definition of pregnancy outcomes with IVF treatment

IVF pregnancy indicators were collected based on three progressive phases: (1) chemical pregnancy (confirmed by blood sample β-human chorionic gonadotropin hormone (β-HCG) levels of above 30 IU/L, which is typically found in the blood of pregnant women as early as 10 days after conception), (2) ongoing pregnancy (above 10 weeks), and (3) live birth (ongoing pregnancy above 24 weeks). All participants were required to have routine medical check-ups after IVF treatment to track these outcomes. Once participants were pregnant (i.e., chemical pregnancy was confirmed), data were recorded for ongoing pregnancy above 10 and 24 weeks.

### Statistical analyses

Descriptive statistics, including estimated means and standard deviations for continuous variables as well as percentages and frequencies for categorical variables, were tabulated. The p values for continuous data were computed with the use of a nonparametric Wilcoxon test. Pearson’s chi-squared test was used for dichotomous categorical data. The odds ratios with 95% confidence intervals (CIs) for IVF treatment outcomes at each stage are reported. Because some participants contribute repeated measurements (i.e., multiple cycles of IVF treatment), additional sensitivity analyses were performed with the use of multivariable mixed effect models adjusted for predefined baseline covariates: age, infertility duration, and pregnancy loss. The ability of the logistic mixed effect regression models to discriminate between the success and failure of IVF treatment was evaluated according to the AUCs (Area under the Receiver Operating Characteristic Curve; ROC). The larger the AUC, the more accurate the prediction model. In the case of perfect prediction, the area beneath will equal 1.0, while an area of 0.5 reflects random forecasts. A larger AUC indicates a more accurate prediction model. An AUC of 1 indicates perfect prediction and an AUC of 0.5 reflects random forecasts [19]. All statistical tests were 2-sided, with a *p*-value of less than 0.05 considered to indicate statistical significance. All analyses were performed using statistical software R 3.3.1 for Windows.

## Results

Table 1 shows the characteristics of study participants stratified by the outcomes of IVF treatment (i.e., chemical pregnancy, ongoing pregnancy, live birth). The mean maternal age was about 36 years. Across different IVF treatment outcomes, the participants with IVF pregnancy were on average younger than those without pregnancy (*p* < 0.01). The average infertility duration was about 4 years. The most common reason for infertility was male factor. In general, the participants with a history of at least one pregnancy loss (i.e., pregnancy loss = “yes”) had relatively higher rates of being not pregnant (i.e., the rates of failure for chemical pregnancy, ongoing pregnancy, and live birth were 30%, 28%, and 27%, respectively). There is a trend of higher HRV values measured at different points of time (i.e., HRV_D, HRV_BO, HRV_BET, HRV_PET) among the IVF pregnancies (i.e., chemical pregnancy, ongoing pregnancy, live birth) as compared to those with IVF treatment failure. The differences in HRV values between different points of time when HRV was measured are also shown in Table 1. Compared to participants without live birth, those having live birth had higher HRV values measured after ET relative to those measured at the first date of menstruation (HRV_D_PET: mean HRV difference between after ET (HRV_PET) and the first date of menstruation (HRV_D): 0.46 vs. -0.49 for patients with live birth and those without live birth, respectively; *p* = 0.033).

**Table 1:** Study participants’ characteristics stratified by pregnancy outcomes with IVF treatment.

Total n = 261 cycles of IVF treatment	Level	n	Chemical pregnancy			Ongoing pregnancy (>10 weeks)			Live birth (>24 weeks)		
Characteristic			Failure (n = 120)	Success (n = 91)		Failure (n = 122)	Success (n = 74)		Failure (n = 128)	Success (n = 66)	
			Mean(sd) or %	Mean(sd) or %	*p*-value	Mean(sd) or %	Mean(sd) or %	*p*-value	Mean(sd) or %	Mean(sd) or %	*p*-value
Age (years)		222	36.77(4.38)	35(4.08)	<0.01	36.7(4.5)	34.44(3.57)	<0.01	36.67(4.45)	34.21(3.51)	<0.01
Gravida		254	0.73(1.07)	0.54(0.76)	0.549	0.71(1.06)	0.54(0.8)	0.607	0.71(1.05)	0.5(0.79)	0.369
Parity		254	0.22(0.49)	0.21(0.41)	0.831	0.23(0.5)	0.2(0.4)	0.904	0.24(0.5)	0.18(0.39)	0.503
Pregnancy loss		250	0.48(0.86)	0.36(0.68)	0.454	0.46(0.85)	0.36(0.69)	0.636	0.44(0.84)	0.34(0.69)	0.571
Infertility duration (years)		248	4.45(3.55)	4.06(2.82)	0.670	4.4(3.49)	3.96(2.89)	0.527	4.49(3.56)	3.71(2.63)	0.279
Infertility factors	Both	254	10%	12%	0.216	11%	12%	0.555	11%	12%	0.702
	Female		18%	21%		21%	22%		20%	23%	
	Male		51%	37%		49%	39%		48%	39%	
	Unknown		20%	30%		20%	27%		20%	26%	
Gravida	0	254	59%	58%	0.932	60%	59%	0.995	59%	62%	0.680
	≥1		41%	42%		40%	41%		41%	38%	
Parity	0	254	81%	79%	0.781	79%	80%	0.948	78%	82%	0.530
	≥1		19%	21%		21%	20%		22%	18%	
Pregnancy loss	0	250	70%	73%	0.581	72%	74%	0.770	73%	75%	0.678
	≥1		30%	27%	0.115	28%	26%	0.573	27%	25%	0.657
HRV indexes											
HRV_D		85	1.54(1.53)	1.88(1.38)	0.109	1.73(1.53)	1.81(1.42)	0.573	1.72(1.49)	1.83(1.47)	0.657
HRV_BO		100	1.88(1.64)	2.31(2.14)	0.478	1.90(1.79)	2.23(1.99)	0.409	1.84(1.75)	2.35(2.03)	0.197
HRV_BET		215	1.74(1.67)	2.58(2.67)	0.013	2.06(2.24)	2.29(2.27)	0.198	2.11(2.25)	2.19(2.3)	0.515
HRV_PET		206	1.60(1.9)	1.54(1.35)	0.440	1.43(1.51)	1.58(1.46)	0.468	1.39(1.4)	1.55(1.38)	0.388
Difference in HRV values											
HRV_D_BO		46	0.22(1.43)	0.98(2.63)	0.858	0.55(1.85)	0.64(2.49)	0.490	0.51(1.83)	0.71(2.56)	0.665
HRV_D_BET		60	0.05(1.8)	0.9(3.19)	0.294	0.02(1.89)	1.12(3.41)	0.198	0.08(1.86)	1.13(3.56)	0.275
HRV_D_PET		59	0(2.95)	0.05(2.39)	0.517	-0.48(2.48)	0.37(2.52)	0.055	-0.49(2.41)	0.46(2.62)	0.033
HRV_BO_BET		83	0.17(2.6)	0.32(3.34)	0.455	0.32(2.8)	0.3(3.17)	0.428	0.38(2.71)	0.21(3.31)	0.144
HRV_BO_PET		79	-0.48(1.43)	-0.64(2.2)	0.673	-0.7(1.67)	-0.5(1.95)	0.846	-0.71(1.65)	-0.5(1.98)	0.916
HRV_BET_PET		202	-0.15(2.49)	-1.09(2.57)	0.098	-0.66(2.63)	-0.74(2.04)	0.714	-0.75(2.53)	-0.67(2.07)	0.877

Abbreviations: HRV: heart rate variability, HRV_D: HRV measured at first date of menstruation, HRV_OB: HRV measured at r-HCG (Ovidrel) administration, HRV_BET: HRV measured before embryo transfer (ET), HRV_PET: HRV measured after ET, HRV_D_BO: difference in HRV value between HRV_D and HRV_OB (i.e., HRV_OB minus HRV_D), HRV_D_BET: difference in HRV value between HRV_D and HRV_BET (i.e., HRV_BET minus HRV_D), HRV_D_PET: difference in HRV value between HRV_D and HRV_PET (i.e., HRV_PET minus HRV_D), HRV_BO_BET: difference in HRV value between HRV_BO and HRV_BET (i.e., HRV_BET minus HRV_BO), HRV_BO_PET: difference in HRV value between HRV_BO and HRV_PET (i.e., HRV_PET minus HRV_BO), HRV_ BET_PET: difference in HRV value between HRV_BET and HRV_PET (i.e., HRV_PET minus HRV_BET),

**Fig 1:**
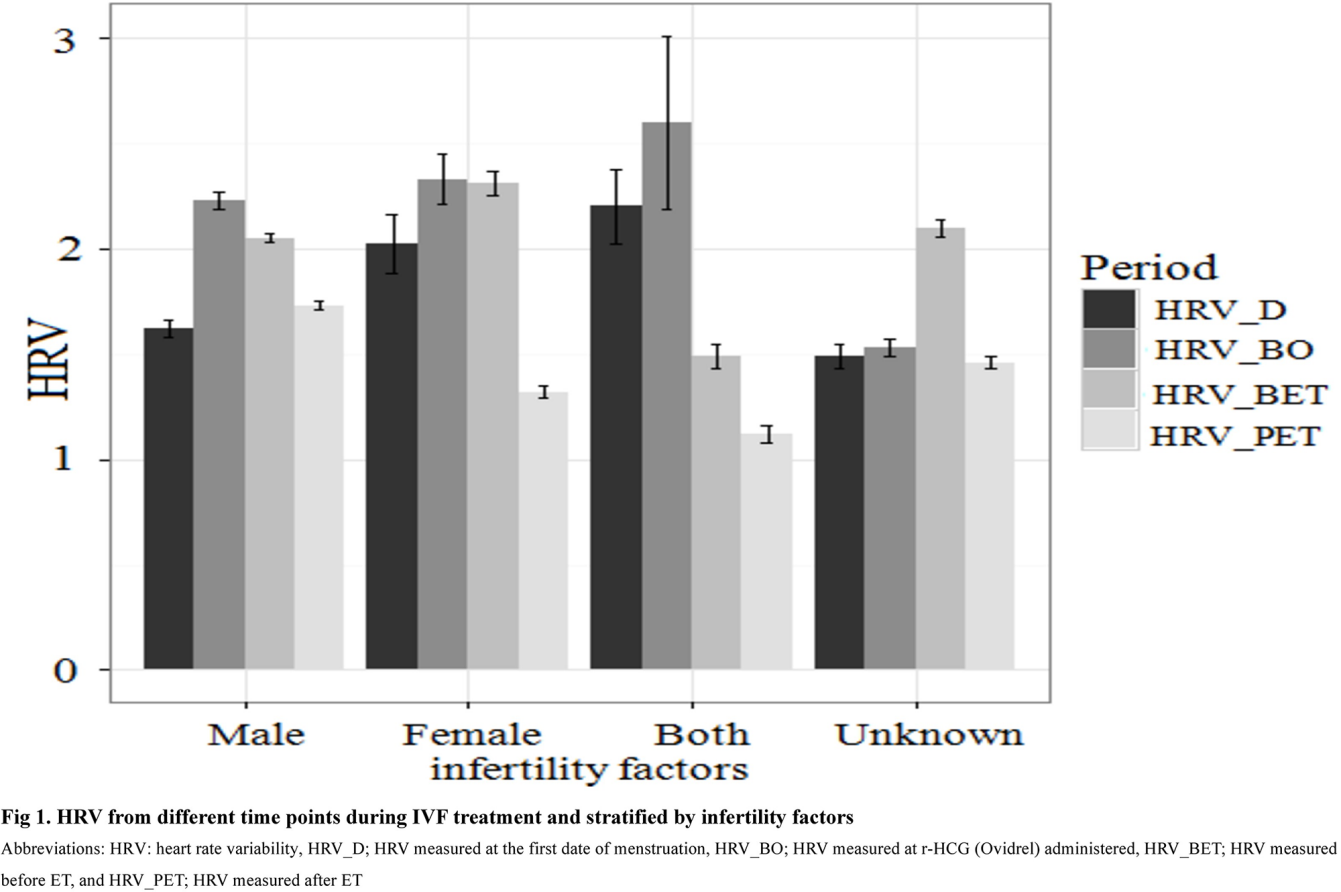
HRV measured at various time points during IVF treatment and stratified by infertility factor.

Fig 1 shows the HRV index values for patients stratified by different infertility factors (i.e., male infertility only, female infertility only, both male and female infertility) and measured at different points of time during IVF treatment (i.e., HRV_D, HRV_OB, HRV_BET, HRV_PET). The HRV values increased during IVF treatment (i.e., from HRV_D to HRV_BO) and decreased after ET (i.e., from HRV_BET to HRV_PET). A significant drop in HRV value from before ET (i.e., HRV_BET) to after ET (i.e., HRV_PET) was observed among patients with female infertility (“Female”) and those with unknown infertility (“Unknown”) (*p* < 0.05).

A comparison across patients with different infertility factors indicates that those with both male and female infertility (“Both”) had the highest HRV values, followed by those with female infertility (“Female”) and then male infertility (“Male”). We further assessed pregnancy rates for patients with different infertility factors and found that patients with “Both” had the best pregnancy outcomes, followed by those with “Female” and then “Male”. Specifically, the percentages of chemical pregnancy were 48%, 46%, and 35% for patients with “Both”, “Female”, and “Male” infertility factors, respectively. The percentages of ongoing pregnancy were 40%, 38%, and 33% for patients with “Both”, “Female”, and “Male” infertility factors, respectively. The percentages of live birth were 36%, 32%, and 29% for patients with “Both”, “Female”, and “Male” infertility factors, respectively. These results show that the patients with higher HRV (i.e., patients with “Both” infertility factor) had better pregnancy outcomes and those with lower HRV (i.e., patients with “Male” infertility factor) had poor pregnancy outcomes with IVF treatment.

**Fig 2:**
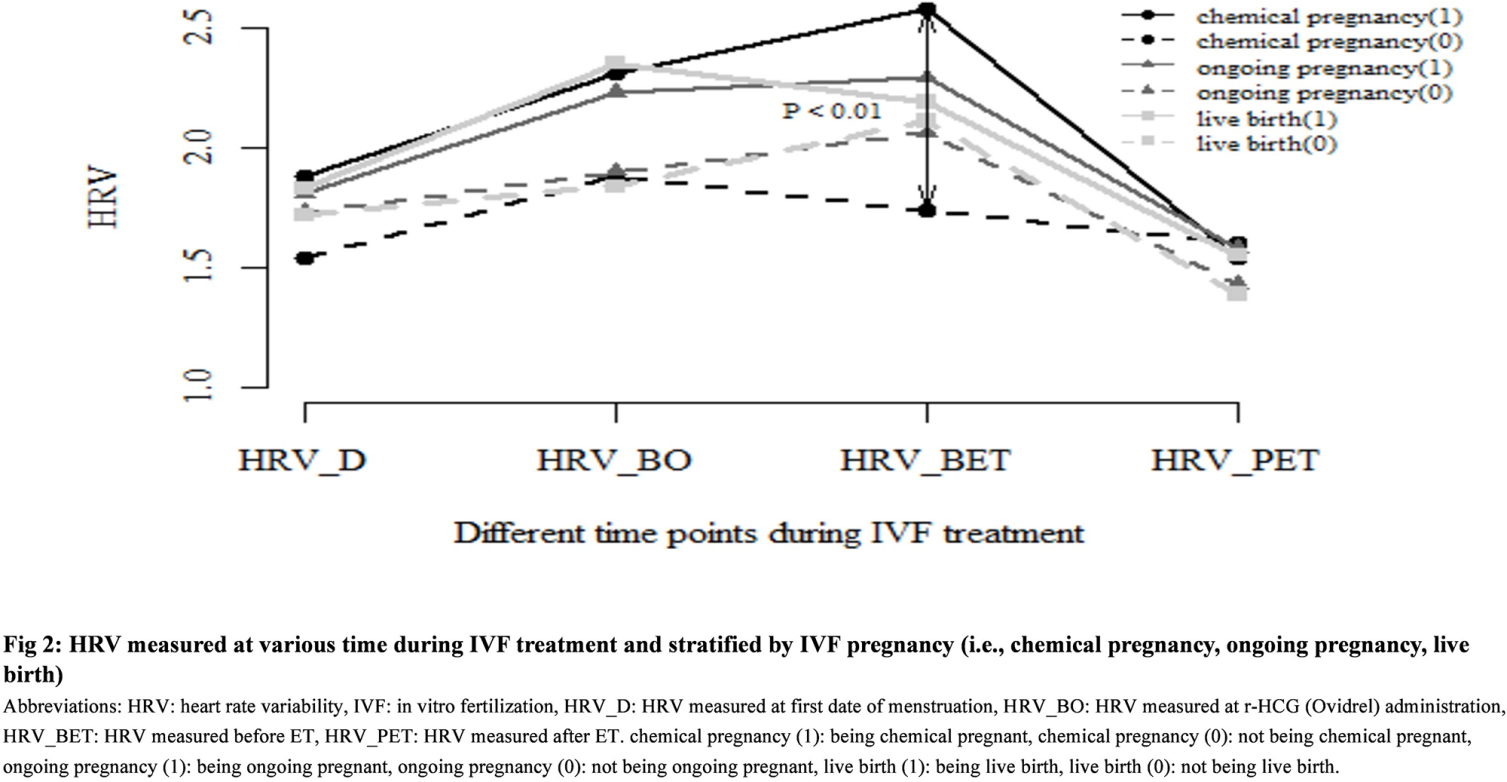
HRV measured at various time points during IVF treatment and stratified by IVF pregnancy (i.e., chemical pregnancy, ongoing pregnancy, live birth).

In Fig 2, we stratified the patients by IVF pregnancy (i.e., chemical pregnancy, ongoing pregnancy, live birth) and analyzed their HRV values measured at the four points of time during IVF treatment (i.e., HRV_D, HRV_BO, HRV_BET, HRV_PET). Similar to the trend shown in Fig 1, the HRV values increased with IVF treatment and then decreased after ET across different pregnancy subgroups in Fig 2. When we analyzed the change in the HRV values at the four points of time of IVF treatment, the change in a trend of HRV values over time was statistically significant in patients with chemical pregnancy and those with live birth (the trend line of HRV values for patients with chemical pregnancy: *p* < 0.01, that of HRV values for patients with live birth: *p* = 0.02). Moreover, women with IVF pregnancies (i.e., chemical pregnancy, ongoing pregnancy, live birth) had a significantly higher average HRV value (the average of HRV_D, HRV_BO, HRV_BET, and HRV_PET values) as compared to that of women without pregnancies (i.e., no chemical pregnancy, no ongoing pregnancy, no live birth) (*p* < 0.05), with the highest value observed in the women with chemical pregnancy. As shown in Fig 2, the HRV before ET (i.e., HRV_BET) was significantly higher in patients with chemical pregnancy as compared to that in those without chemical pregnancy (*p* < 0.01).

**Fig 3:**
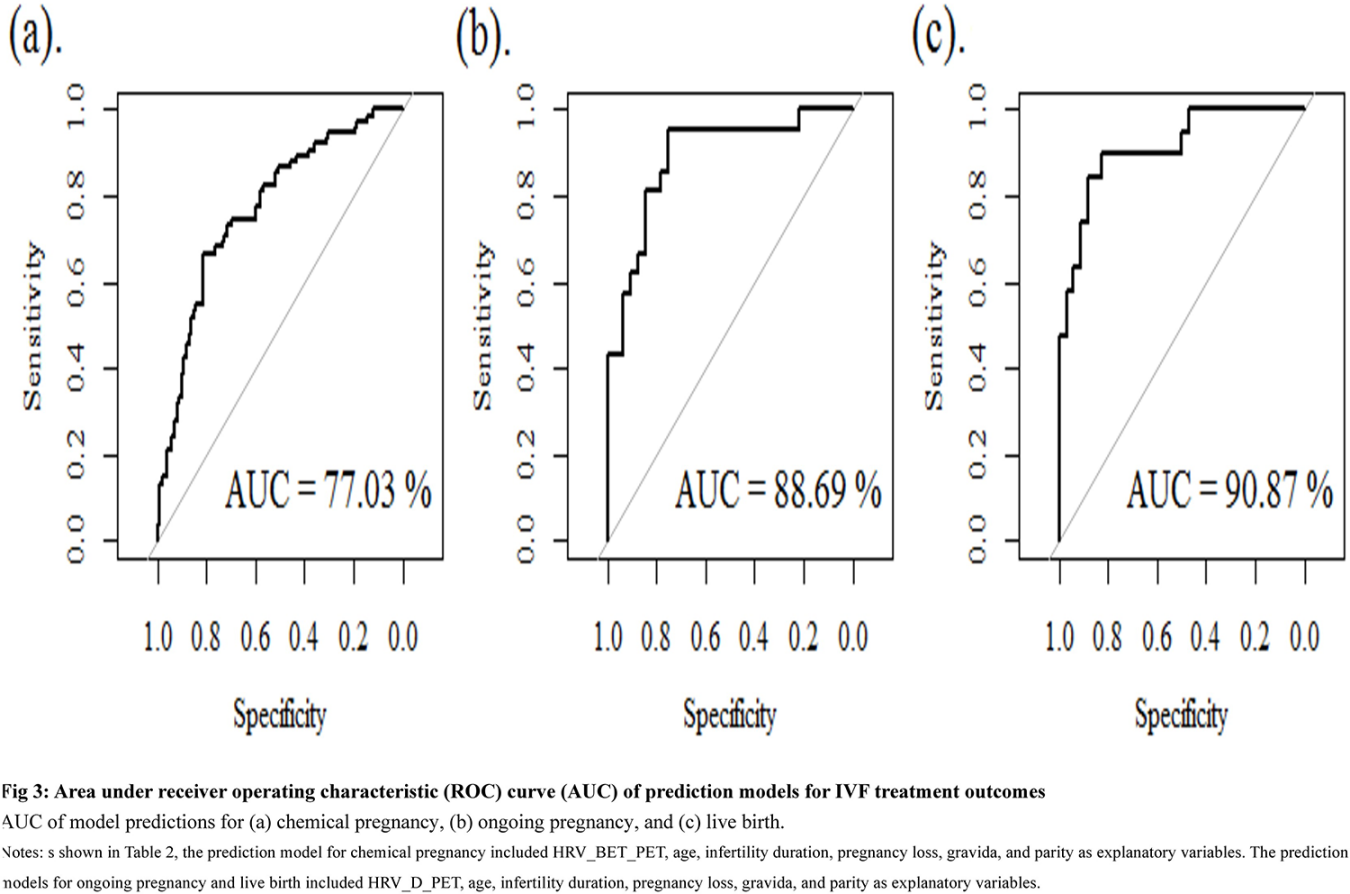
Area under receiver operating characteristic (ROC) curve (AUC) of prediction models for IVF treatment outcomes.

The results from the mixed effect model analysis for assessing the association between individual variables and IVF pregnancy indicators are provided in Supplementary Table 1. We selected only statistically significant individual variables from the mixed effect model analyses for the multivariate mixed effect models (in Table 2). We found that maternal age was the most significant factor across different pregnancy outcome models (i.e., chemical pregnancy, ongoing pregnancy, live birth). For a one year increase in maternal age, the odds of pregnancy decreased by 16% (odds ratio, OR: 0.84, 95% CI: 0.76–0.93, *p* < 0.01), 25% (OR: 0.75, 95% CI: 0.58–0.93, *p* = 0.02), and 28% (OR: 0.72, 95% CI: 0.54–0.91, *p* = 0.01) for chemical pregnancy, ongoing pregnancy, and live birth, respectively. Among HRV indices, only the difference in HRV before and after ET (i.e., HRV_BET_PET) was statistically significantly associated with chemical pregnancy. With a one unit increase in HRV change before and after ET, the odds of chemical pregnancy decreased by 18% (OR: 0.82, 95% CI: 0.70–0.97, *p* < 0.02). We further examined the predictive ability of these multivariate models (in Table 2) for IVF pregnancy outcomes by using the AUC. As shown in Fig 3, the AUCs were 0. 77 (95% CI: 0.70, 0.84), 0.89 (0.79, 0.98), and 0.91(0.83, 0.99) for the models of chemical pregnancy, ongoing pregnancy, and live birth, respectively, implying satisfactory model prediction.

**Table 2:** Results of multivariate mixed effect models for IVF pregnancy outcomes.

Variable	Chemical pregnancy (Number of patients: 177, Number of IVF cycle: 142)		Ongoing pregnancy (>10 weeks) (Number of patients: 53, Number of IVF cycle: 50)		Live birth (>24 weeks) (Number of patients: 53, Number of IVF cycle: 50)	
	OR (95% C.I.)	*p*-value	OR (95% C.I.)	*p*-value	OR (95% C.I.)	*p*-value
HRV_BET_PET	0.82(0.70–0.97)	0.02*				
HRV_D_PET			1.18(0.85–1.64)	0.33	1.23(0.82–1.83)	0.32
Maternal age	0.84(0.76–0.93)	<0.01**	0.75(0.58–0.93)	0.02*	0.72(0.54–0.91)	0.01*
Infertility duration	0.96(0.85–1.08)	0.50	1.11(0.83–1.50)	0.47	1.13(0.81–1.57)	0.47
Pregnancy loss (>0 vs. 0 = ref)	0.40(0.10–1.55)	0.18	0.65(0.02–20.88)	0.81	1.92(0.04–93.58)	0.74
Gravida (>0 vs. 0 = ref)	4.09(0.82–20.38)	0.09	7.96(0.10–625.76)	0.35	4.11(0.03–567.88)	0.57
Parity (>0 vs. 0 = ref)	0.44(0.12–1.63)	0.22	0.16(0–5.97)	0.32	0.13(0–11.08)	0.37

Abbreviations: IVF: in vitro fertilization treatment, OR: odds ratio, C.I.: confidence interval, HRV: heart rate variability, HRV_BET_PET: difference in HRV value before and after ET, HRV_D_PET: difference in HRV value between first date of menstruation and time when r-HCG (Ovidrel) was administered.

* *p* < 0.05,

** *p* < 0.01

## Discussion

To our best knowledge, this is the first study to measure the HRV index during IVF treatment and to investigate the predictive ability of HRV for pregnancy outcomes with IVF treatment. This study extends existing knowledge on the clinical value of the HRV index to the field of female fertility. The results are of importance for the care of women undergoing IVF treatment.

HRV is an important indicator of psychological health, general and cardiovascular health, and mortality [20–22]. Patients presenting with anxiety or depressive disorders are likely to have relatively low HRV values [23]. Undergoing IVF treatment is typically stressful and causes a lot of psychological distress to patients [1–3]. As observed in this study for patients undergoing IVF treatment, the low HRV, which may be contributed by increased level of psychological distress, was likely to be associated with poor pregnancy outcomes of IVF treatment. We found that women without IVF pregnancy had lower HRV values across four different points during IVF treatment compared to those of women with IVF pregnancies (in Fig 2). These results imply that a relatively high level of HRV, an indication of healthy autonomic and cardiovascular responses, may be associated with better pregnancy outcomes of IVF treatment (e.g., chemical pregnancy). In contrast, low or reduced HRV values indicate that the sympathetic and parasympathetic nervous systems may not be properly coordinating to provide an appropriate heart rate response, which may be contributed by increased anxiety or depressive symptoms which occur during stressful procedures of IVF treatment. In fact, a Japanese study has found that increased psychological distress (measured by the Kessler 6 scale [24]) and decreased HRV indices were observed in those with unexplained pregnancy loss (especially in those with pregnancy loss ≧ 3) [15]. Also, previous studies have shown that altered autonomic nervous system activity during pregnancy may be associated with adverse uterine environment and subsequent worse pregnancy outcomes [15, 25]. These findings might explain our observation of relatively low HRV values among women without IVF pregnancies.

Moreover, women with a “Male” infertility factor were found to have low HRV (Fig 1), which may explain their low rate of IVF pregnancies. This highlights a critical need for clinical attention and further interventions to explore physical or psychological stressors for such women. Tailored strategies (e.g., consultants, stress management programs, anxiety reduction treatment) should be developed and delivered during the period of IVF treatment, in particular for the women with relatively low HRV.

HRV is a commonly used measure for cardiac autonomic regulation and provides insight into both health and disease [17]. The HRV index has become a strong diagnostic biomarker for general health as well as specific diseases (e.g., cardiovascular diseases) [17]. However, it is not commonly used during IVF treatment. The measures of HRV are an easily accessible window into autonomic activity. HRV analysis is a low-cost, non-invasive, and simple method that reflects the balance of the autonomic nervous system regulation of the heart rate and offers the opportunity to detect the presence of autonomic dysfunction complicating several illnesses [26, 27]. This study suggests that HRV as a proxy of cardiac autonomic regulation could be a potential prognostic tool for the care of patients undergoing IVF treatment. In particular, we found that the difference in the HRV index before and after ET was a significant predictive factor for chemical pregnancy; a larger difference indicates lower chance of chemical pregnancy (Table 2). However, the HRV index for more progressive phases of pregnancy (i.e., ongoing pregnancy, live birth) may be less predictive, which may however be due to the limited sample size of this study or unadjusted confounders or risk factors during pregnancy. Nevertheless, these results imply that the difference or change in HRV values (i.e., before and after ET), rather than an absolute HRV value from a certain point of time during IVF treatment, is predictive for pregnancy outcomes with IVF treatment, highlighting the importance of tracking and closely monitoring HRV changes during IVF treatment to provide prognostic prediction for pregnancy.

The effect of maternal age on pregnancy outcomes with IVF treatment has been previously investigated. Higher maternal age leads to poorer outcomes with IVF treatment [28], especially in women over 40 years old [29]. Consistently, we found that maternal age at the time of IVF was the most significant predictor across different IVF pregnancy outcome indicators (i.e., chemical pregnancy, ongoing pregnancy, live birth). Advanced maternal age had negative correlations with the outcomes of IVF treatment. The consistent findings from this study with previous research [28, 29] support the validity of our results.

Some limitations of this study need to be acknowledged. First, no mental health assessment (e.g., using self-reported questionnaires or interviewing) was applied in this study. Second, all participants were from one medical center in southern Taiwan, with predominant participants with male infertility factor (45%). So, selection bias in the present study could not be eliminated. And, our findings may be applicable to only a subset of ethnically Chinese women. Ethnically Chinese people are distributed over a large geographic area, and are likely to have differences in dietary habits, physical activity, and even treatment approaches. Third, this study did not record the detail of anthropometric data and the usage of drugs for study participants, and autonomic reactivity tests which typically comprise deep breathing, valsalva maneuver and head-up tilt were also not performed. Fourth, there is the lack of statistical power to detect the association between the HRV index and the outcomes of ongoing pregnancy and live birth, in part due to limited follow-up study cases for these two outcomes (i.e., the number of patients: 53 and the number of IVF cycle: 50 in the models for ongoing pregnancy and live birth outcomes, Table 2). Fifth, we only analyzed for the LF/HF ratio, an index for sympatho-vagal balance, which however may not represent the respiratory sinus arrhythmia or overall variability in heart rate. Lastly, we did not apply sample size estimation in such an observational study. This is in part because the present study is the first research to explore the relationship between HRV measures and IVF pregnancies; there is no prior expectation or previous evidence on the association between HRV measures and IVF pregnancy, which are however essential information for sample size calculation.

We hope the preliminary results from this study to guide planning future large-scale research. The future research with a large number of IVF-EF cycles is needed to clarify the association between HRV and IVF treatment outcomes and also include the data of SDNN and/or total power (i.e., VLF+LF+HF) in analysis. And, whether HRV measures among women with IVF treatment are associated with mental symptoms (e.g., anxiety, depression) is needed to be confirmed in the future studies.

## Conclusions

The results of present study suggest that the changes in HRV measures during IVF treatment may serve as prognostic predictors of IVF pregnancy. This highlights the clinical value of HRV measures in the care of women undergoing IVF treatment and suggests that coping strategies or interventions (e.g., consultations) can be tailored based on the HRV values measured during IVF treatment to improve the pregnancy rates of these patients.

## Supporting information

S1 Table Results of simple mixed effect models for IVF pregnancy outcomes. (DOCX)
