# Correction: Heart rate variability among women undergoing *in vitro* fertilization treatment: Its predictive ability for pregnancy

**DOI:** 10.1371/journal.pone.0196880

**Published:** 2018-05-01

**Authors:** Meng-Hsing Wu, Pei-Fang Su, Kuan-Ya Chen, Tung-Hee Tie, Hsu-Cheng Ke, Hau Chen, Yu-Chi Su, Yu-Chen Su, Huang-Tz Ou

There are errors in the author affiliations. The affiliations should appear as shown here:

Meng-Hsing Wu^1^, Pei-Fang Su^2^, Kuan-Ya Chen^2^, Tung-Hee Tie^3^, Hsu-Cheng Ke^3^, Hau Chen^3^, Yu-Chi Su^3^, Yu-Chen Su^3^, Huang-Tz Ou^4,5,6^

**1** Department of Obstetrics and Gynecology, College of Medicine and Hospital, National Cheng Kung University, Tainan, Taiwan, **2** Department of Statistics, National Cheng Kung University, Tainan, Taiwan, **3** School of Medicine, College of Medicine, National Cheng Kung University, Tainan, Taiwan, **4** Institute of Clinical Pharmacy and Pharmaceutical Sciences, College of Medicine, National Cheng Kung University, Tainan, Taiwan, **5** Department of Pharmacy, College of Medicine, National Cheng Kung University, Tainan, Taiwan, **6** Department of Pharmacy, National Cheng Kung University Hospital, Tainan, Taiwan.
