# Supplementary material for: Heart rate variability among women undergoing in vitro fertilization treatment: Its predictive ability for pregnancy
Source: PLoS One. 2018 Mar 12;13(3):e0193899. doi: 10.1371/journal.pone.0193899 (PMC5846774; doi:10.1371/journal.pone.0193899)
Supplement: S1 Table — (DOCX) [file pone.0193899.s001.docx]

**S1 Table: Results of simple mixed effect models for IVF pregnancy outcomes**

| Total n = 261 |  |  | Chemical pregnancy | |  | Ongoing pregnancy (>10 weeks) | |  | Live birth (>24 weeks) | |
| --- | --- | --- | --- | --- | --- | --- | --- | --- | --- | --- |
| Characteristic | Level | n | OR ( 95% C.I. ) | *p*-value | n | OR ( 95% C.I. ) | *p*-value | n | OR ( 95% C.I. ) | *p*-value |
| Age (years) |  | 198 | 0.9(0.83-0.97) | 0.01* | 183 | 0.84(0.74-0.95) | 0.01* | 181 | 0.81(0.68-0.95) | 0.01* |
| Gravida |  | 209 | 0.79(0.57-1.1) | 0.16 | 194 | 0.82(0.58-1.16) | 0.26 | 192 | 0.76(0.52-1.13) | 0.18 |
| Parity |  | 209 | 0.93(0.49-1.78) | 0.83 | 194 | 0.85(0.42-1.73) | 0.65 | 192 | 0.69(0.31-1.53) | 0.36 |
| Pregnancy loss |  | 205 | 0.8(0.54-1.2) | 0.28 | 190 | 0.85(0.55-1.31) | 0.45 | 188 | 0.82(0.51-1.33) | 0.43 |
| Infertility duration (years) |  | 203 | 0.96(0.87-1.06) | 0.46 | 188 | 0.95(0.85-1.07) | 0.41 | 186 | 0.91(0.8-1.03) | 0.15 |
| Infertility factors | Both | 209 |  |  | 194 |  |  | 192 |  |  |
|  | Female |  | 0.93(0.32-2.75) | 0.90 |  | 0.92(0.28-3.01) | 0.89 |  | 1.02(0.29-3.58) | 0.97 |
|  | Male |  | 0.60(0.22-1.6) | 0.30 |  | 0.70(0.24-2.06) | 0.52 |  | 0.74(0.23-2.32) | 0.60 |
|  | Unknown |  | 1.20(0.42-3.40) | 0.74 |  | 1.20(0.38-3.82) | 0.76 |  | 1.14(0.33-3.94) | 0.83 |
| Gravida | 0 | 209 |  |  | 194 |  |  | 192 |  |  |
|  | ≥1 |  | 1.05(0.58-1.9) | 0.87 |  | 1.04(0.54-2) | 0.92 |  | 0.89(0.44-1.79) | 0.73 |
| Parity | 0 | 209 |  |  | 194 |  |  | 192 |  |  |
|  | ≥1 |  | 1.09(0.53-2.24) | 0.82 |  | 0.97(0.43-2.16) | 0.94 |  | 0.75(0.32-1.79) | 0.52 |
| Pregnancy loss | 0 | 205 |  |  | 190 |  |  | 188 |  |  |
|  | ≥1 |  | 0.86(0.44-1.68) | 0.66 |  | 0.95(0.44-2.02) | 0.89 |  | 0.88(0.39-2) | 0.76 |
| HRV indexes |  |  |  |  |  |  |  |  |  |  |
| HRV_D |  | 67 | 1.18(0.83-1.69) | 0.35 | 63 | 1.05(0.72-1.52) | 0.81 | 63 | 1.06(0.73-1.55) | 0.76 |
| HRV_BO |  | 83 | 1.13(0.89-1.44) | 0.31 | 80 | 1.1(0.86-1.41) | 0.44 | 80 | 1.16(0.9-1.5) | 0.25 |
| HRV_BET |  | 193 | 1.22(1.04-1.43) | 0.02* | 178 | 1.04(0.9-1.21) | 0.58 | 176 | 1.01(0.86-1.19) | 0.87 |
| HRV_PET |  | 187 | 0.97(0.81-1.17) | 0.78 | 172 | 1.08(0.86-1.36) | 0.5 | 170 | 1.14(0.85-1.52) | 0.39 |
| Difference in HRV values |  |  |  |  |  |  |  |  |  |  |
| HRV_D_BO |  | 41 | 1.22(0.85-1.74) | 0.28 | 41 | 1.02(0.74-1.4) | 0.91 | 41 | 1.05(0.76-1.44) | 0.77 |
| HRV_D_BET |  | 58 | 1.15(0.91-1.45) | 0.23 | 54 | 1.21(0.92-1.59) | 0.17 | 54 | 1.19(0.91-1.54) | 0.2 |
| HRV_D_PET |  | 57 | 1.01(0.83-1.23) | 0.94 | 53 | 1.17(0.9-1.52) | 0.25 | 53 | 1.19(0.91-1.55) | 0.21 |
| HRV_BO_BET |  | 77 | 1.02(0.87-1.19) | 0.82 | 74 | 1(0.84-1.18) | 0.98 | 74 | 0.98(0.83-1.16) | 0.8 |
| HRV_BO_PET |  | 76 | 0.95(0.74-1.22) | 0.70 | 73 | 1.07(0.81-1.41) | 0.63 | 73 | 1.07(0.81-1.42) | 0.62 |
| HRV_BET_PET |  | 183 | 0.85(0.74-0.98) | 0.02* | 168 | 0.99(0.86-1.14) | 0.9 | 166 | 1.02(0.87-1.19) | 0.81 |

Abbreviations: HRV: heart rate variability, HRV_D: HRV measured at first date of menstruation, HRV_OB: HRV measured at r-HCG (Ovidrel) administration, HRV_BET: HRV measured before embryo transfer (ET), HRV_PET: HRV measured after ET, OR: odds ratio, C.I.: confidence interval, HRV_D_BO: difference in HRV value between HRV_D and HRV_OB (i.e., HRV_OB minus HRV_D), HRV_D_BET: difference in HRV value between HRV_D and HRV_BET (i.e., HRV_BET minus HRV_D), HRV_D_PET: difference in HRV value between HRV_D and HRV_PET (i.e., HRV_PET minus HRV_D), HRV_BO_BET: difference in HRV value between HRV_BO and HRV_BET (i.e., HRV_BET minus HRV_BO), HRV_BO_PET: difference in HRV value between HRV_BO and HRV_PET (i.e., HRV_PET minus HRV_BO), HRV_ BET_PET: difference in HRV value between HRV_BET and HRV_PET (i.e., HRV_PET minus HRV_BET),

* *p* < 0.05, ** *p* < 0.01, , *** *p* < 0.001
